# Dengue Versus Multisystem Inflammatory Syndrome – When the Grey Zone Gets Thinner

**DOI:** 10.7759/cureus.20276

**Published:** 2021-12-08

**Authors:** Aneesh Basheer, Nadeemu Rahman, Eldho George, Manish Murali

**Affiliations:** 1 General Medicine, DM Wayanad Institute of Medical Sciences, Wayanad, IND

**Keywords:** covid-19, adults, thrombocytopenia, inflammation, dengue

## Abstract

Despite the decline in COVID-19 cases, the potential threat of multisystem inflammatory syndrome (MIS) - a hyperinflammatory response following acute SARS-CoV-2 infection - looms large. Although initially described in children, it is being increasingly reported in adults. In dengue endemic regions, MIS is likely to cause diagnostic dilemma with dengue due to overlapping clinical and laboratory features. We describe a young male with fever, gastrointestinal symptoms, a transient rash, thrombocytopenia, and positive dengue NS1 antigen test. Early onset of thrombocytopenia, significant gastrointestinal symptoms and protracted fever were atypical, raising suspicion of MIS-A in view of a recovery from a recent SARS-CoV-2 infection. However, absence of neurologic and cardiac manifestations, stable hemodynamics, absence of mucosal involvement and negative inflammatory markers helped in managing the patient conservatively as dengue. This case highlights diagnostic challenges faced by clinicians treating suspected dengue in the face of increasing recognition of MIS and need for systematic research to establish diagnostic criteria for MIS-A.

## Introduction

Dengue is endemic in many parts of the world including India. Arrival of monsoons often coincides with a spike in cases. Previously, diagnosis of dengue in such areas was straightforward, supported by positive serological tests in a patient with typical clinical features. However, COVID-19 and the multisystem inflammatory syndrome (MIS) that share many features with dengue have made the clinical diagnosis and management complex, posing new challenges. Our patient had recovered from COVID-19 two months back and presented with high-grade fever, malaise, gastrointestinal symptoms and thrombocytopenia. A positive NS1 antigen test strengthened the clinical likelihood of dengue while the transient rash, unduly protracted fever and gastrointestinal symptoms raised suspicion of MIS. Careful monitoring of blood pressure, use of inflammatory markers and serial blood counts helped avert the unnecessary use of steroids in our case, as patient became better by eighth day of illness.

## Case presentation

A 22-year-old man with no previous comorbidities presented to the outpatient department with high-grade fever for one day. It was associated with severe malaise and joint pains involving all large joints. Besides patient had nausea, vomiting, diarrhea and abdominal pain. He had been staying away from home along with a friend who had fever a week back and had tested positive for dengue. He also had a history of COVID-19 two months back that was treated at home symptomatically. He was completely asymptomatic since then. At the time of presenting to us, he had no history of rashes, soreness of mouth and tongue or bleeding manifestations. The history of dengue fever in his friend strengthened the likelihood of dengue from common environmental exposure. At the same time, a recovery from COVID-19 with prominent gastrointestinal, rheumatological and dermatological symptoms made it difficult to exclude multisystem inflammatory syndrome with sufficient degree of certainty. Absence of mucosal involvement, however, pointed against multisystem inflammatory syndrome. On examination, he was alert, cooperative and febrile. Blood pressure was 110/70 mmHg and pulse rate was 90 beats/minute. He was dehydrated. Lymph nodes were not palpable. Systemic examination was unremarkable. The oral cavity and tongue were normal. On the third day of illness we noticed erythematous rash over the trunk and intense redness of eyes that disappeared by the next morning. High-grade fever persisted until the eighth day of illness.

**Figure 1:**
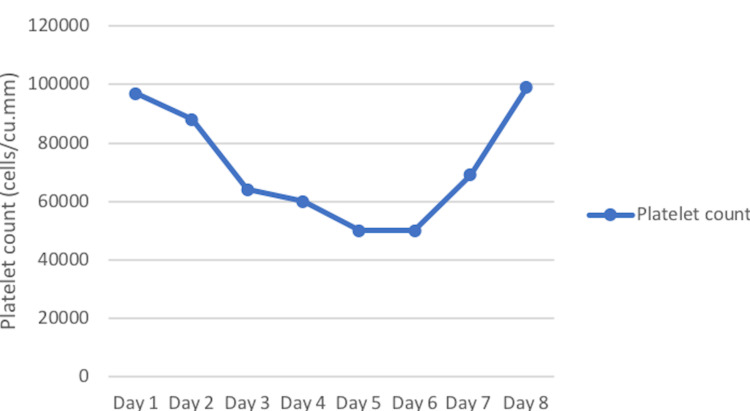
Trends in platelet count during the course of illness Platelet counts are in cells per cubic mm.

The initial complete blood count showed hemoglobin of 13.8 g/dL (normal: 12-15 g/dL), hematocrit of 41% (normal: 42-45%), total leucocyte count of 7800 cells/cu.mm (normal: 4000-11000/cu.mm) and normal differential counts. The platelet count was 97,000/cu.mm and showed a progressive fall over the course of illness until sixth day (Figure 1). Real time polymerase chain reaction (RT-PCR) and rapid antigen test for SARS-CoV-2 were negative. Liver functions, renal functions and serum lipase were within normal limits. Dengue non-structural protein 1 (NS1) antigen was positive on day 2 of the illness. Since the clinical suspicion of multisystem inflammatory syndrome was moderately high, C-reactive protein and serum ferritin were performed. Both were normal.

While the primary working diagnosis was dengue, possibility of multisystem inflammatory syndrome could not be ruled out given the recent recovery from COVID and manifestations involving multiple organs like joints, gastrointestinal tract, rash, conjunctival suffusion and thrombocytopenia. The following points favored dengue: short febrile illness, history of dengue in a friend sharing the same environment during the same time, thrombocytopenia and positive Dengue NS1 antigen. The odd points that brought in a differential diagnosis of multisystem inflammatory syndrome include high-grade fever persisting beyond seven days, an early drop in platelet counts (on day 1 itself), prominent gastrointestinal symptoms including diarrhea and recent recovery from COVID-19. However, lack of hypotension throughout the course of illness, a normal C-reactive protein and ferritin along with absence of mucosal lesions (oral cavity and eyes) were reassuring.

We administered intravenous fluids (normal saline) to correct dehydration and titrated the fluid replacement using the daily hematocrit as the trigger. We aimed to maintain hematocrit within normal range by upscaling fluids whenever it rose and reducing flow rates when it dropped. Fever was managed symptomatically with oral paracetamol. Ondansetron and Pantoprazole were administered intravenously during the first three days in view of nausea and vomiting. During the febrile period when the platelet counts were on the decline, we discussed in detail with the patient about the possibility of multisystem inflammatory syndrome and why we thought this might only be a second possibility. We informed the patient that our plan was to watch for any fall in blood pressure and/or new mucosal lesions to initiate steroids which are indicated for multisystem inflammatory syndrome, and also the potential harms of starting them empirically at that point when the diagnosis of dengue was more likely. The patient agreed to stick to this plan after informed weighing of benefits and risks.

Twice daily monitoring of hematocrit and platelet counts, meticulous blood pressure monitoring and daily enquiry about bleeding manifestations provided reassuring trends. Platelet count showed an initial decline and recovered by sixth day of illness. Patient became afebrile on the eighth day with remarkable improvement in systemic symptoms. He was seen two weeks later when he had no fever and normal blood counts. He had resumed college by then.

## Discussion

As early as April 2020, multisystem inflammatory syndrome was described in a group of children as a sequel of COVID-19 [1]. All affected children had fever, rash, gastrointestinal symptoms and shock. Markers of inflammation such as C-reactive protein and ferritin were elevated [1]. Subsequently a large series of 570 children with MIS-C was reported from the United States [2]. It is believed to represent an immune response following recovery from SARS-CoV-2 infection. It might also represent persistence of SARS-CoV-2 infection outside the respiratory tract. Altered regulation of the Renin-angiotensin-aldosterone axis and widespread endothelial damage with inflammation and thrombosis have been proposed as additional mechanisms [3]. In June 2020, cases of hyperinflammatory state resembling MIS-C were reported in adults. A report in October 2020 described 16 cases of multisystem inflammatory syndrome in adults (MIS-A) characterized by fever, mucositis, gastrointestinal symptoms, cardiac dysfunction and shock [4]. Although not as frequent as in children, MIS-A cases are being diagnosed and reported increasingly.

In Southeast Asia and India dengue is endemic and the presenting features including fever, rash, thrombocytopenia and joint pain overlap considerably with those of MIS-A. In addition, reports of dengue and COVID-19 co-infection have also been reported from several countries [5,6]. Further, a significant reduction in reported dengue cases during 2020 suggests that dengue cases might have been underdiagnosed due to focus on detection of COVID-19 in endemic regions [7]. With the increasing reports of MIS-A, clinicians now face the challenge of distinguishing dengue from MIS-A. Some studies in children have found younger age and higher levels of inflammatory markers such as CRP to be associated with MIS-C while abdominal pain and erythematous rash were more common in dengue [8]. However, no studies have examined this in adults.

Currently, the diagnosis of MIS-A is suspected in young adults with shock, thrombocytopenia and elevated inflammatory markers on a background of diverse clinical presentations such as fever, rash, non-purulent conjunctivitis, neurologic dysfunction, cardiac involvement (myocarditis, pericarditis, heart blocks or ventricular tachycardia) or gastrointestinal symptoms. Since our knowledge of MIS-A is still evolving, no uniform diagnostic criteria have been laid down. Our patient was a young adult in his twenties, with a history of recent recovery from COVID-19 and was currently tested negative by antigen test and RT-PCR. His presentation with high-grade fever that lasted beyond seven days, prominent gastrointestinal symptoms, rash, non-purulent conjunctivitis and early thrombocytopenia from first day of illness raised suspicion despite a positive dengue NS1 antigen test. In general, febrile phase in dengue seldom lasts beyond seven days and thrombocytopenia becomes evident from third day reaching a nadir by 5-6 days and restoration by day 7 or 8 [9,10]. The differentiation of dengue from MIS-A becomes critical since the latter has a remarkable response to parenteral steroids and intravenous immunoglobulin which may be lifesaving in those with shock and cardiac dysfunction.

In our patient, the lack of cardiac involvement and neurological involvement was against MIS-A. The patient was normotensive throughout hospital stay and inflammatory markers were negative. These increased our confidence in pursuing a watchful approach and deferring the use of steroids or intravenous immunoglobulin. However, we ensured meticulous hemodynamic monitoring and serial monitoring of blood counts in order to offer these treatments early should any fall in blood pressure occur.

Given the complex diagnostic and treatment issues pertaining to dengue and MIS-A, more research is needed to determine clinical and laboratory determinants that might serve to differentiate the two. While the paucity of data precludes issuance of any guidelines for diagnosis and management of MIS-A as of now, limitations in dengue endemic regions must be taken into account as and when such recommendations are developed in future.

## Conclusions

Dengue shares many clinical and diagnostic features with multisystem inflammatory syndrome. Atypical manifestations in dengue patients such as protracted high-grade fever, prominent gastrointestinal symptoms, rash, non-purulent conjunctivitis and early onset thrombocytopenia must raise suspicion of multisystem inflammatory syndrome in patients who have recovered recently from COVID. Careful monitoring of blood pressure, daily examination for new features such as mucosal lesions and rashes could help detect multisystem inflammatory syndrome and start steroids or intravenous immunoglobulin in timely manner. Normal inflammatory markers like C-reactive protein may be reassuring in such circumstances.
